# All-Inorganic CsPbBr_3_ Perovskite Films Prepared by Single Source Thermal Ablation

**DOI:** 10.3389/fchem.2020.00313

**Published:** 2020-04-21

**Authors:** Lucia Nasi, Davide Calestani, Francesco Mezzadri, Fabrizio Mariano, Andrea Listorti, Patrizia Ferro, Marco Mazzeo, Roberto Mosca

**Affiliations:** ^1^IMEM - CNR Institute of Materials for Electronics and Magnetism, Parma, Italy; ^2^Department of Chemistry, Life Sciences and Environmental Sustainability, University of Parma, Parma, Italy; ^3^CNR NANOTEC, Institute of Nanotechnology, Lecce, Italy; ^4^Dipartimento di Matematica e Fisica “Ennio De Giorgi”, Università del Salento, Lecce, Italy; ^5^Department of Chemistry, University of Bari “Aldo Moro”, Bari, Italy

**Keywords:** halide perovskite, cesium lead tribromide, single source, vacuum deposition, green electroluminescence

## Abstract

Hybrid organo-lead halide perovskites are becoming the benchmark material for next generation photovoltaics and a very important player for other applications such as photodetectors and light emitting diodes. Nevertheless, the most important issue hindering the large-scale application of these materials remains their intrinsic instability due to the organic cation. Although the substitution with inorganic cesium (Cs) enhances stability, in most cases solution deposition methods of fully inorganic perovskites result in high surface roughness and poor surface coverage. This work reports on the evaporation of the CsPbBr_3_ precursor by Single Source Thermal Ablation, showing that just after deposition films consist of a mixture of CsPbBr_3_, CsPb_2_Br_5_, and Cs_4_PbBr_6_ due to a vertical composition gradient. We point out that mild post deposition treatments lead to the conversion of CsPb_2_Br_5_ and Cs_4_PbBr_6_ into CsPbBr_3_ due to its higher thermodynamic stability. Conversion results into smooth and pinhole-free CsPbBr_3_ films with good light absorption and emission properties. We demonstrate the suitability of obtained films for planar devices by preparing perovskite-based pure-green light emitting diodes, thus promoting Single Source Thermal Ablation as a promising alternative deposition technique for all-inorganic perovskite-based devices.

## Introduction

In the last few years, the introduction of metal halide perovskites in solution processable solar cells led to impressive advances that today make perovskite solar cells (PCSs) the fastest developing solar technology (Kumar Jena et al., 2019). Perovskite-based light emitting diodes (PeLEDs), also impressed for their rapid and effective rise (Lin et al., 2018). The extraordinary speed of these advances is at the origin of the enormous attention devoted in the last decade to the metal halide perovskite family.

Until now, the best performing perovskites (ABX_3_) include in the A-site organic cations such as methylammonium (MA) and formamidinium (FA), which make these materials suffer from environmental degradation under moisture, oxygen, heat, and illumination exposure, so that long-term stability remains the main issue hindering perovskite real-life application (Wang et al., 2016; Zhao et al., 2019). Substituting the organic cations with inorganic cesium (Cs) enhances device stability for both I-(Swarnkar et al., 2016) and Br-based perovskites (Kulbak et al., 2015). In particular, CsPbBr_3_ combines improved stability (Kulbak et al., 2015) with interesting features, such as direct bandgap, high electron mobility, and long carrier lifetime (Stoumpos et al., 2013), which make this material interesting for not only solar cells but also photodetectors (Song et al., 2016), visible light communications (Dursun et al., 2016), LEDs (Cho et al., 2017).

Several methods, including spin coating of precursor solutions in one step (Zhang et al., 2017; Cheng et al., 2019) or by sequential deposition (Kulbak et al., 2016), have been investigated in order to obtain a dense and uniform perovskite CsPbBr_3_ layer. However, these approaches often result in discontinuous film morphologies, with a high pinholes density, mainly due to the low solubility of the CsBr precursor in commonly employed solvents. Consequently, device performances may be severely limited by bad interfaces and electrical shunt paths resulting from high surface roughness and poor surface coverage. The use of additives has been proposed to improve film uniformity (Zhang et al., 2017; Lin et al., 2018; Cheng et al., 2019), but achieving a complete surface coverage remains a difficult task. Moreover, wet deposition techniques are hardly compatible with hetero-structure architectures, since the number of allowed layers is limited by their solubility in orthogonal solvents, thus limiting the possibility of device engineering.

Compared to solution-processing methods, vacuum evaporation not only provides uniform and compact films with high reproducibility and fine thickness control, but also allows the fabrication of multi-layer structures of thin films without chemical modifications of the underlying layers thus enabling the fabrication of hetero-junction devices with enhanced performances. Most of the works on vacuum-based methods for ABX_3_ halide perovskites use the dual source evaporation method (Sessolo et al., 2015; Ono et al., 2016; Mariano et al., 2017), that was first applied to halide perovskites by evaporating simultaneously MAI and PbCl_2_ precursors for the deposition of planar solar cells (Liu et al., 2013). Later on, dual source evaporation has been used to demonstrate not only efficient planar PSCs based on CsPbI_3_ (Malinkiewicz et al., 2014; Chen et al., 2017; Frolova et al., 2017), CsPbBr_3_ (Lei et al., 2018), and CsPbIBr_2_ (Ma et al., 2016; Chen et al., 2017), but also CsPbBr_3_ PeLEDs (Hu et al., 2017) and lasers (Zhang L. et al., 2018). However, this method has distinct disadvantages mainly related to the use of significantly different vapors that makes keeping control over the precursor ratio difficult.

An alternative to dual source evaporation is the single source vapor deposition (SSVD) technique where powders of either raw precursors or preformed perovskites are placed into an alumina thermal source that is heated by a rapid increase of the work current. This method was used to evaporate CsPbX_3_ (X = Cl, Br, and I) perovskites (El Ajjouri et al., 2018), but results achieved were not as successful as those reported for MAPbI_3_ (Fan et al., 2016). In particular, for X = Br, as prepared films were made of a mixture of CsPbBr_3_ and CsPb_2_Br_5_, the latter being the dominant phase. CsPbBr_3_ films were obtained only upon annealing evaporated films, but photoluminescence (PL) was lost after the thermal treatment, thus making these films not suitable for light emitting devices.

Flash evaporation is another alternative approach to the vacuum deposition of halide perovskite films, and is faster than SSVD as the evaporation process takes a few seconds instead of a few minutes (Fan et al., 2016). Usually, it is based on a single thermal source consisting in a metal foil heater used to evaporate instantly the perovskite by passing a large current. This simple method was used for organometal halide perovskites by Mitzi et al. (2001) who named it as single source thermal ablation (SSTA) technique. In the era of halide perovskites for PVs, only few papers reported the deposition of three dimensional halide perovskites by flash evaporation (Longo et al., 2015; Xu et al., 2016), all dealing with MAPbI_3_. Due to the high vapor pressure of MAI, high quality films require not only high currents to make MAI and PbI_2_ evaporation as simultaneous as possible, but also a MAI excess in the precursor (up to MAI to PbI_2_ molar ratio of 2.0) (Xu et al., 2016) in order to compensate the inevitable loss of MAI during the deposition process. Since also the preparation of the precursor into the heater plays a role on the film quality, setting up the deposition to achieve high quality films may be not straightforward, so that single source thermal ablation is often considered poorly reproducible if compared to dual source evaporation. A different situation may be expected when MA is replaced by cesium because the vapor pressures of Cs halides are a few orders of magnitudes lower than that of methylammonium halides. To the best of our knowledge, flash evaporation of Cs-based halide perovskites was only recently reported (Tai et al., 2019), but using a technique named “flash-evaporation printing” (FEP). FEP is less simple than SSTA since it exploits a laser beam as the heating source and carbon nanotube sheets as the evaporator onto which perovskite solution is spin coated. In that case, only stoichiometric CsPbI_2_Br films were achieved, while the properties of obtained films were discussed only after annealing, so that film formation remained unclear.

Here we report on flash evaporation CsPbBr_3_ precursor layer by single source thermal ablation. We demonstrate that CsPb_2_Br_5_ and Cs_4_PbBr_6_ phases originated by an unavoidable vertical composition gradient, turn into CsPbBr_3_ either spontaneously in low humidity or upon mild post-deposition thermal treatments, thus resulting into smooth and pinhole-free films with good light absorption and emission properties. These features make the obtained CsPbBr_3_ films very interesting for heterostructure planar devices such as tandem solar cells or PeLEDs. The actual exploitation of flash-evaporated films for device applications is assessed through the fabrication of PeLEDs that showed a very narrow pure green emission. Reported results allow SSTA to be envisaged as a vacuum deposition technique suitable for all-inorganic perovskites.

## Experimental

A 0.45 M precursor solution was prepared in a nitrogen-filled glove box by dissolving at room temperature equimolar amounts of CsBr (Aldrich) and PbBr_2_ (Aldrich) in DMSO under continuous stirring. The test tube was then sealed and removed from the glove box. In order to improve reproducibility, the subsequent operations were performed in rooms where temperature was maintained in the 19–22°C range and relative humidity (RH) was kept below 20%. Hereafter, these environmental conditions will be referred as “DH atmosphere.”

Glass substrates were cleaned by sequential rinsing in 1% Hellmanex, de-ionized water, hot acetone, and isopropanol for 15 min. After drying in blowing nitrogen, up to four substrates were placed on a sample holder that was mounted into the evaporation chamber at a fixed vertical distance of 8 cm from the evaporation source. The evaporation system was tailor made (Mosca et al., 2011) following the schematization given by Mitzi et al. (2001) and allows power to be increased from 0 to the preset value with a rate of ~400 W/s, maximum power being 1,000 W.

The precursor layer for evaporation was created by spreading 40 μl of the precursor solution onto a tantalum boat (Testbourne S46-0.005Ta) that was then placed on a hotplate at 80°C covered by a Petri dish. Temperature was then raised to 100°C to promote DMSO evaporation. This procedure was repeated by adding another 40 μl of the precursor solution. As the final step, the boat was heated at 120°C for 5 min to remove the excess solvent, finally producing a pale orange layer (inset in Figure S1) that was checked by powder XRD, showing the sole presence of CsPbBr_3_ crystallized in the orthorhombic perovskite structure (Figure S1) (Rodová et al., 2003). This confirms the quality of the precursor material, being consistent with the 1:1 PbBr_2_/CsBr molar ratio of the parent solution.

The boat with the precursor layer was then clamped between two electrodes in the evaporation chamber that was immediately pumped to vacuum. When the pressure in the chamber was below 2 × 10^−5^ mbar, electrical current was passed through the boat. For a preset power of 200 W the deposition process was accomplished in about 4 s, but power was shut down after a few more seconds, i.e., as soon as the boat becomes incandescent, so as to prevent precursor residues from remaining in the boat. After waiting 60 s in vacuum, argon was finally inlet and samples removed from the chamber. Unless stated otherwise, films were stored into a dark dry box.

Under these conditions, a film thickness of 250 nm was measured by a standard stylus profilometer. Fifty and one hundred nanometer thick films were obtained for PeroLED by using 34 and 34 + 34 μl, respectively, and increasing the distance between boat and substrates to 12 cm in order to improve thickness uniformity. Thirty nanometer thick films aimed at TEM characterization were prepared by using 21 μl solution with a distance of 12 cm.

When aiming to investigate the evolution of the evaporation process, deposition was performed in two steps: in the former one, 150 W power was applied until the precursor layer was seen to start evaporating (step 1). The chamber was then opened, substrates were replaced by virgin others and the chamber was pumped again to vacuum. Then, a second evaporation was performed by applying 150 W power until the boat became incandescent (step 2).

Thermal annealing treatments were performed into a laboratory oven in DH atmosphere.

All the film characterizations were carried out in DH atmosphere. In particular, the structural quality of the films was studied by X-ray diffraction (XRD) measurements in a Siemens (D500) powder diffractometer, with CuK_α_ radiation. Diffraction patterns were collected by 0.05° 2θ steps and counting times of 5 s per step. In some cases, counting times as low as 2 s per step were used in order to minimize measurement duration despite an increase of the background noise.

Film absorbance spectra were measured by a Jasco UV–vis V-530 spectrometer. Morphological characterization was performed by atomic force microscopy (AFM) using a Veeco Dimensions 3100 SPM and by scanning electron microscopy (SEM) using a Zeiss Auriga field emission microscope (FESEM) operated at 5 kV. Selected Area Electron Diffraction (SAED) and High-Resolution transmission Electron Microscopy (HREM) were performed in a Jeol 2200FS Transmission Electron Microscope (TEM).

Optical Properties Steady state and time resolved photoluminescence were measured by an Edinburgh FLS920 spectrometer equipped with a Peltier- cooled Hamamatsu R928 photomultiplier tube (185–850 nm). An Edinburgh Xe900 450 W Xenon arc lamp was used as exciting light source. Corrected spectra were obtained via a calibration curve supplied with the instrument (lamp power in the steady state PL experiments 0.6 mW cm^−2^, spot area 0.5 cm^2^). Emission decay time were determined with the single photon counting technique by means of the same Edinburgh FLS980 spectrometer using a laser diode as excitation source (1 MHz, exc = 635 nm, 67 ps pulse width and about 30 ps time resolution after deconvolution) and a Hamamatsu MCP R3809U-50 (time resolution 20 ps) as detector (Laser power in the TRPL experiment 1.6 Wcm^−2^, spot area 0.3 mm^2^) (Masi et al., 2018).

Concerning PeLEDs fabrication, before the deposition of the organic compounds, Indium Tin Oxide (ITO)-covered glass substrates were cleaned in acetone, isopropanol, and deionized water for 10 min at 60°C in an ultrasonic bath. On top of the oxide anode, the hole injection film consists of 40 nm of spin-coated poly(3,4-ethylenedioxythiophene) polystyrene sulfonate (Pedot:PSS). Then, the n-doped layer, following CsPbBr_3_ films, consisted of 50 nm of 4,7-diphenyl 1,10-phenanthroline (BPhen) doped with cesium atoms. A 10 nm thick neat film of pure BPhen after the emissive layer was also included as hole-blocking layer. The structure was closed with a cathode of 120 nm thick silver (Ag).

The small molecules materials were deposited by thermal evaporation in a Kurt J. Lesker organic thin film and metallization deposition system, at a base pressure around 10^−8^ mbar.

The fabricated PeLEDs have been characterized by electrical-optical measurements performed under vacuum with an Optronics OL770 spectrometer, coupled, through an optical fiber, to the OL610 telescope unit for the luminance measurements. The whole system, NIST calibrated using a standard lamp, was directly connected by RS232 cable to a Keithley 2420 current-voltage source meter.

## Results and Discussion

The films studied in this work were obtained by single source thermal ablation of a layer that was prepared on the Ta boat by using an equimolar solution of CsBr and PbBr_2_ in DMSO, as explained in the Experimental methods. Soon after the deposition, films obtained by this kind of precursor layers exhibit a full coverage of the substrate (Figure S2). AFM measurements indicate a RMS roughness of 5.6 nm, meaning that films are smooth, as expected for good quality evaporated films. Moreover, AFM images point out that films are made of extended areas similar to large grains connected to each other. Unfortunately, those areas could not be studied by SEM-EDS or TEM investigations because films were unstable under electron irradiation.

**Figure 1:**
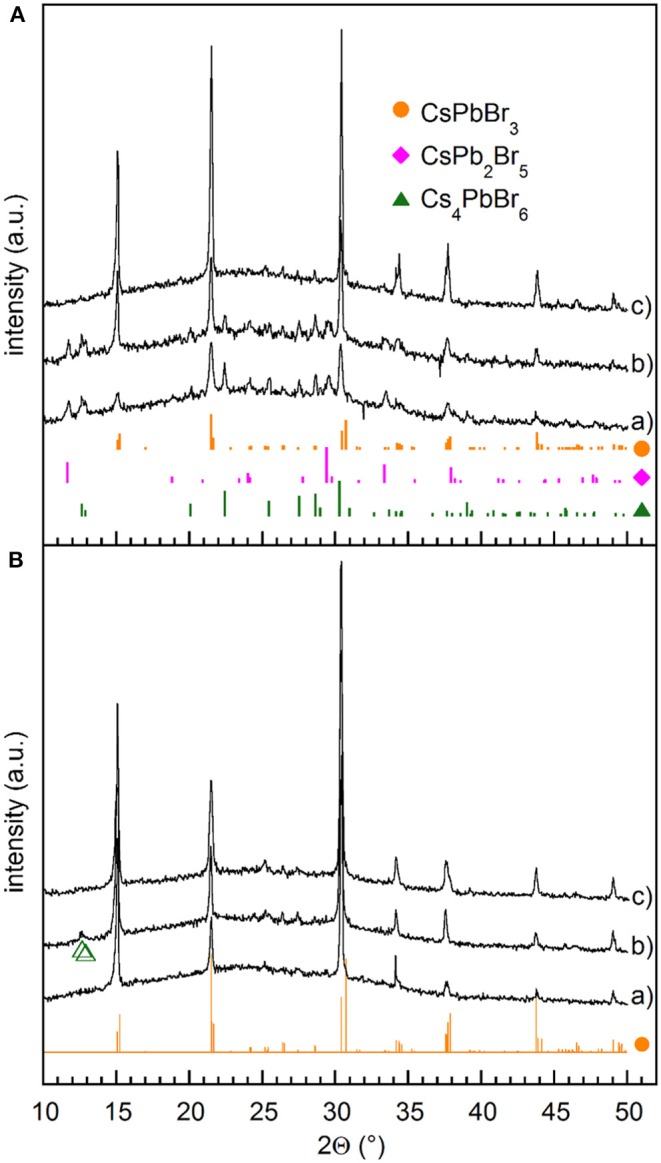
**(A)** XRD patterns measured on films (a) as deposited, (b) stored in vacuum for 10 days, (c) stored in vacuum for 90 days. **(B)** XRD patterns measured on films (a) stored in DH atmosphere (RH≤20%) for 24 h, (b) annealed at 100°C for 90 min immediately after the removal from the evaporation chamber, and (c) annealed at 100°C for 60 min after overnight storage in DH atmosphere (RH≤20%). Green empty triangles correspond to Cs_4_PbBr_6_. The reference patterns of CsPbBr_3_ (•), CsPb_2_Br_5_ (♦), and Cs_4_PbBr_6_ (▴) are also shown.

Powder XRD patterns collected immediately after removing the sample from the evaporation chamber (Figure 1, pattern a) point out the presence of not only orthorhombic CsPbBr_3_ (Rodová et al., 2003), but also other peaks that we identified as corresponding to bulk tetragonal CsPb_2_Br_5_ (ICSD254290) and rhombohedral Cs_4_PbBr_6_ (ICSD 254272). Noteworthy, obtained films show pronounced, although partial (010), orientation of the crystallites, likely induced kinetically by the growth conditions.

The coexistence of CsPbBr_3_ with both CsPb_2_Br_5_ and Cs_4_PbBr_6_, may appear rather surprising because they are generally regarded as PbBr_2_-rich and CsBr-rich phases, respectively, and usually CsPbBr_3_ is found to coexist with either the former (Zhang et al., 2016) or the latter (Wu C. et al., 2017). A mixture of CsPbBr_3_ and CsPb_2_Br_5_ was found in films prepared by SSVD from preformed CsPbBr_3_ precursors (El Ajjouri et al., 2018), which suggests that vacuum evaporation comes with a partial decomposition of the perovskite precursor. In order to clarify the origin of the observed phase mixture in films obtained from a pure CsPbBr_3_ precursor phase, deposition was split into two steps that allowed us to analyze the products of the initial and final evaporation stages separately, as explained in the Experimental methods. XRD measurements performed after each step (Figure S3) point out that the initial stages of the evaporation process give films composed by only CsPb_2_Br_5_, whilst a mixture of CsPbBr_3_ and Cs_4_PbBr_6_ is deposited subsequently. Therefore, film composition is expected to change from PbBr_2_-rich near the substrate to CsBr-rich near the film surface, so that the XRD pattern (a) reported in Figure 1A does not come from a real mixture of the three phases, but from a composition gradient through the film thickness. Likewise, in the flash evaporation of MAPbI_3_, MAI starts evaporating before PbI_2_ after the initial perovskite decomposition (Xu et al., 2016). In order to make MAI and PbI_2_ evaporation as simultaneous as possible, heating time was reduced by increasing the current passing through the boat. In the present case, even a power as high as 800 W gave films with XRD patterns similar to those obtained at 200 W (Figure S4), which means that increasing power does not allow us to achieve as deposited films with homogenous vertical compositions.

Interestingly, we noticed that the structural properties of films change with time at room temperature. Indeed, films stored in vacuum for 10 days exhibit the presence of CsPbBr_3_ with increased crystal size, as pointed out by peak sharpening and residual impurities of CsPb_2_Br_5_ and Cs_4_PbBr_6_ (Figure 1A pattern b), while transformation into CsPbBr_3_ goes to completion after several tens of days (Figure 1A pattern c). In all cases strong (010) preferential orientation is detected, likely induced by the specific kinetics of the growth process. The elemental mapping of the converted films, obtained by TEM/EDXS analysis, exhibits a uniform spatial distribution of Cs, Pb, and Br with Cs:Pb:Br = 19.8:19.2:61, which are very close to the stoichiometric ratio of CsPbBr_3_ (Figure S5). For these films, TEM/SAED patterns and HRTEM confirmed the complete transformation into CsPbBr_3_ phase without lattice defects besides the grain boundaries (Figure S5). Thus the time evolution of films suggests that, despite similar formation energies shown by the different phases of the Cs–Pb–Br family (Zhang Z. et al., 2018), the CsPbBr_3_ phase is the most energetically favorable in films containing equimolar amounts of PbBr_2_ and CsBr.

When films are stored in DH atmosphere, the conversion into CsPbBr_3_ is much faster. Indeed, after 24 h at RH≤20%, XRD measurements reveal only CsPbBr_3_, while features due to either CsPb_2_Br_5_ or Cs_4_PbBr_6_ cannot be observed (Figure 1B pattern a). On the contrary, three phases are still observed in films stored 10 days in a dry box pointing out that moisture plays a significant role in catalyzing the conversion. Transformations between CsPbBr_3_ and CsPb_2_Br_5_ or Cs_4_PbBr_6_ were studied mainly with reference to nanocrystals (Wu L. et al., 2017; Li et al., 2018; Su et al., 2018) and were explained by a water-triggered stripping of CsBr. In the present work, we find that both Cs_4_PbBr_6_ and CsPb_2_Br_5_ spontaneously react in vacuum at room temperature to form CsPbBr_3_, and that the presence of water favors such transformation. This means that water-induced CsBr extraction itself does not explain the observed evolution. The influence of moisture was recently studied on CsPbBr_3_ films obtained by a solution method (Di Girolamo et al., 2019), showing that relatively high humidity (RH = 60%) at first improves CsPbBr_3_ crystalline quality by increasing the mobility of ionic species, but then results in the degradation of CsPbBr_3_ into CsPb_2_Br_5_ and/or Cs_4_PbBr_6_. We note here that low humidity levels considered in the present work favors the conversion of CsPb_2_Br_5_ and Cs_4_PbBr_6_ into CsPbBr_3_. Although the tendency of halide perovskites to suffer from phase transformation and instability, including surface hydration and ion migration, is well-known (Yin et al., 2017), understanding the mechanisms of this transformation deserves further investigation that is out of the scopes of this work.

Conversion into CsPbBr_3_ was also obtained through the decomposition of CsPb_2_Br_5_ by thermal annealing at 220°C (Li et al., 2017), while Cs_4_PbBr_6_ was converted at temperatures above 150°C in vacuum (Palazon et al., 2017). We observed that conversion into CsPbBr_3_ can be achieved by treatments at lower temperatures. Indeed, annealing as-deposited films at 100°C for at least 90 min improves the crystallization of the CsPbBr_3_ phase, while leaving traces of Cs_4_PbBr_6_ (Figure 1B, pattern b), that were detected as a minor phase even after 24 h at 70°C. On the contrary, complete transformation was achieved reproducibly after the overnight storage in DH atmosphere followed by annealing at 100°C for 60 min (Figure 1B, pattern c). Increasing annealing temperatures above 130°C makes complete conversion faster, but produces a crystallographic reorientation (Figure S6), so that the annealed samples show (001) preferential orientation. This reorientation is likely related to the lattice distortion induced by the phase transitions taking place at 88 and 130°C, that transform the crystal structure from orthorhombic to tetragonal and cubic, respectively. At the same time, it is worth noting that no other phases appear when the annealing is prolonged after complete conversion, not even at 200°C.

**Figure 2:**
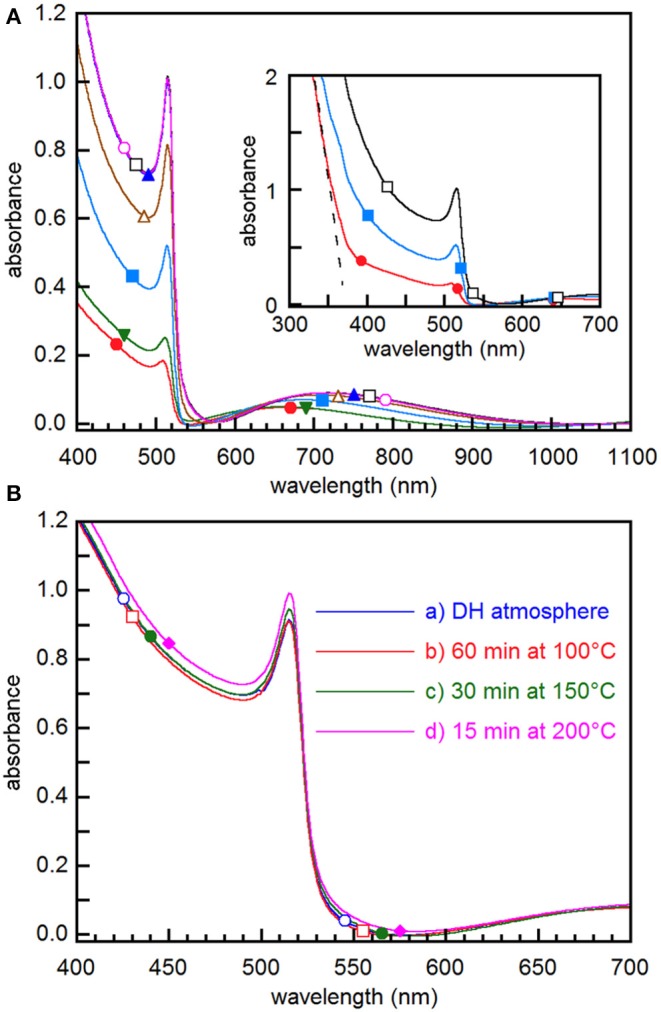
**(A)** Absorbance spectra taken on the same film as removed from the evaporation chamber (•) and then after storage in DH atmosphere for 5 min (▾), 50 min (■), 100 min (▵), 200min (▴), 350 min (□), and 10 days (◦). In the inset, the dashed line points out the steep absorbance increase below 370 nm. **(B)** Absorbance spectra of films (a) stored in DH atmosphere for 24 h, (b) stored overnight in air and then annealed at 100°C for 60 min, (c) annealed at 150°C for 30 min, and (d) annealed at 200°C for 15 min.

UV-vis absorption spectroscopy points out that spectra are modified by film exposure to DH atmosphere (Figure 2A), most of the changes taking place in the first 80 min. All the spectra exhibit a sharp onset at about 530 nm and an excitonic peak at ~515 nm (Figure 2) that are typical of CsPbBr_3_ thin films (Akkerman et al., 2015; Yantara et al., 2015; Lei et al., 2018; Di Girolamo et al., 2019). The broad absorbance peaks in the 650–750 nm range are interference maxima (see Figure S7 and the relevant discussion), whose positions vary during conversion likely due to the influence of the composition change on the refractive index. The presence of interference fringes, while pointing out the good surface quality of the films, does not allow us to determine reliably the bandgap value during the transformation process. However, once conversion has come to an end, the Tauc plot method gives a value of 2.36 eV (Figure S8), which is the bandgap of CsPbBr_3_ (Akkerman et al., 2015; Kulbak et al., 2015; Lei et al., 2018). The height of the absorbance step increases with exposure time, indicating that CsPbBr_3_ content increases in the film, consistently with the XRD measurements results. Through the absorbance at the excitonic peak maximum, we estimate that the initial spectrum comes from about 28% CsPbBr_3_. At 19°C and RH≤20%, the height of the onset increases to 80% of the final value in about 100 min and reaches saturation after about 400 min, while no further spectral evolution is observed after further 10 days aging in dark dry box (Figure 2A), which confirms that converted films are rather stable.

It is worth noting that initial spectra exhibit a second steep absorbance increase below 370 nm (inset of Figure 2A), where the absorption edge due to CsPb_2_Br_5_ is expected (Dursun et al., 2016), thus confirming that such phase is present in the early stages of the conversion. However, the second onset cannot be followed in its time evolution because, as shown by XRD measurements above (Figure 1), the CsPbBr_3_ content in the film increases with time, while CsPb_2_Br_5_ gradually disappears, thus resulting in a predominant light absorption by CsPbBr_3_. No signature of the Cs_4_PbBr_6_ phase could be obtained because, due to the wide bandgap of 3.95 eV (Akkerman et al., 2015), the absorbance step falls in a short wavelength range where light absorption is dominated by CsPbBr_3_ and CsPb_2_Br_5_ even in the as extracted films.

When films are converted by thermal annealing, spectra show the same features as the sample converted by exposure to DH atmosphere for 24 h, differences in treatments affecting to a minor extent the absorbance (Figure 2B). This means that treatments at temperature higher than 130°C, while yielding the crystallographic reorientation of the film, give results similar to those achieved by aging at room temperature, as far as light absorption is concerned. Owing to the excellent substrate coverage and film uniformity pointed out by the large-scale SEM images (Figure S9), from the absorbance we obtained the absorption coefficient through the Lambert-Beer law, achieving a value of ~1.1 × 10^5^ cm^−1^ at 400 nm. This value is comparable with that of CsPbBr_3_ single crystals (Song et al., 2017) and larger than that of nanocrystals (de Roo et al., 2016), thus confirming that films we obtained may be considered as excellent light absorbers.

**Figure 3:**
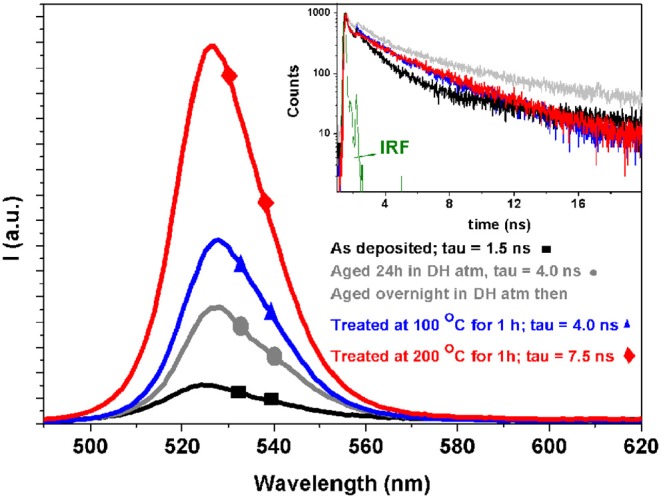
Steady state (main panel) and time resolved (inset) photoluminescence spectra of four different samples. The legend are common in the main panel and in the inset. A stretched exponential analysis is used to evaluate the decays. In green the instrumental response function. For the details of the measurements see the Experimental Section.

In order to further assess the optical properties of the film, we performed steady state and time resolved photoluminescence analysis (Figure 3). PL curves are in line of what usually observed for CsPbBr_3_ perovskite (Zhang et al., 2017), being characterized by a wide, intense emission band in the “Green” spectral region (around 520 nm peak). The decay time associated to this emission band is an indicator of perovskite material suitability for optoelectronic applications (Colella et al., 2016). The observed trend is very clear and reflects the material evolution discussed above, from the as prepared compounds characterized by a shorter living emission toward the long living fully converted CsPbBr_3_ species. The photoluminescence decays follow the emission intensity increase (inset in Figure 3), so that the best performing films, among those considered here, are those treated at 200°C for 60 min. It is worth noting that the only previous work dealing with single source evaporation of all-inorganic halide perovskites (El Ajjouri et al., 2018) showed that PL was quenched by the annealing used to convert films into CsPbBr_3_. The discrepancy with results reported here can be explained considering that precursor decomposition is minimized by SSTA, that is intrinsically faster than SSVD. Thus, the evolution into CsPbBr_3_ may differ in films obtained by the two techniques, so that a lower content of non-radiative recombination centers is provided by SSTA.

**Figure 4:**
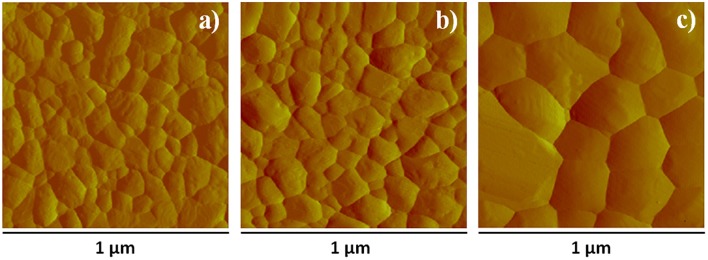
AFM surface amplitude images of films **(a)** aged in DH atmosphere for 24 h, **(b)** annealed at 100°C for 60 min after overnight storage in DH atmosphere, **(c)** annealed at 200°C for 60 min. The corresponding AFM height images are reported in Figure S10.

The above results indicate that CsPbBr_3_ films obtained by SSTA are suitable for PeLED fabrication provided that they have proper morphology. AFM/SEM experiments show that the conversion into CsPbBr_3_ leads to a significant morphology change with respect to as deposited films considered in Figure S2, and that final morphology depends on treatment (Figure 4, Figure S11). Indeed films converted in DH atmosphere point out average grain size of about 100 nm (Figure 4a) and roughness of 5.8 nm, which is not far from the 5.6 nm value measured soon after the removal from the evaporation chamber. Converting the films by thermal annealing at 100°C (for 60 min) results in a very similar surface morphology as the film converted in air (Figure 4b), although grain average size slightly decreases and roughness increases up to 7.8 nm, which indicates that surface remains rather smooth. On the other hand, by increasing the annealing temperature to 200°C, significant changes in surface characteristics are observed, including the increase of the average grain size to ~200 nm (Figure 4c) and roughness to about 30 nm. In addition, grains having micrometric lateral size and as high as 200 nm appear (Figure S11C) and are observed as white spots in the SEM images (Figure S9), where few pinholes show up. These features are likely related to the combined action of grain boundary migration and crystallographic reorientation induced by the phase transitions discussed above. High temperature annealing is then an issue whenever smooth and pinhole-free films are needed. Work is in progress to investigate if short annealing processes similar to those discussed by Tai et al. (2019) may be used profitably to increase grain size while maintaining films smooth and compact.

In the light of the above results, we conclude that, foreseeing device applications, the best trade-off between morphology and optical properties is offered by films where conversion into CsPbBr_3_ is achieved by the overnight storage in air followed by an annealing at 100°C for 60 min.

**Figure 5:**
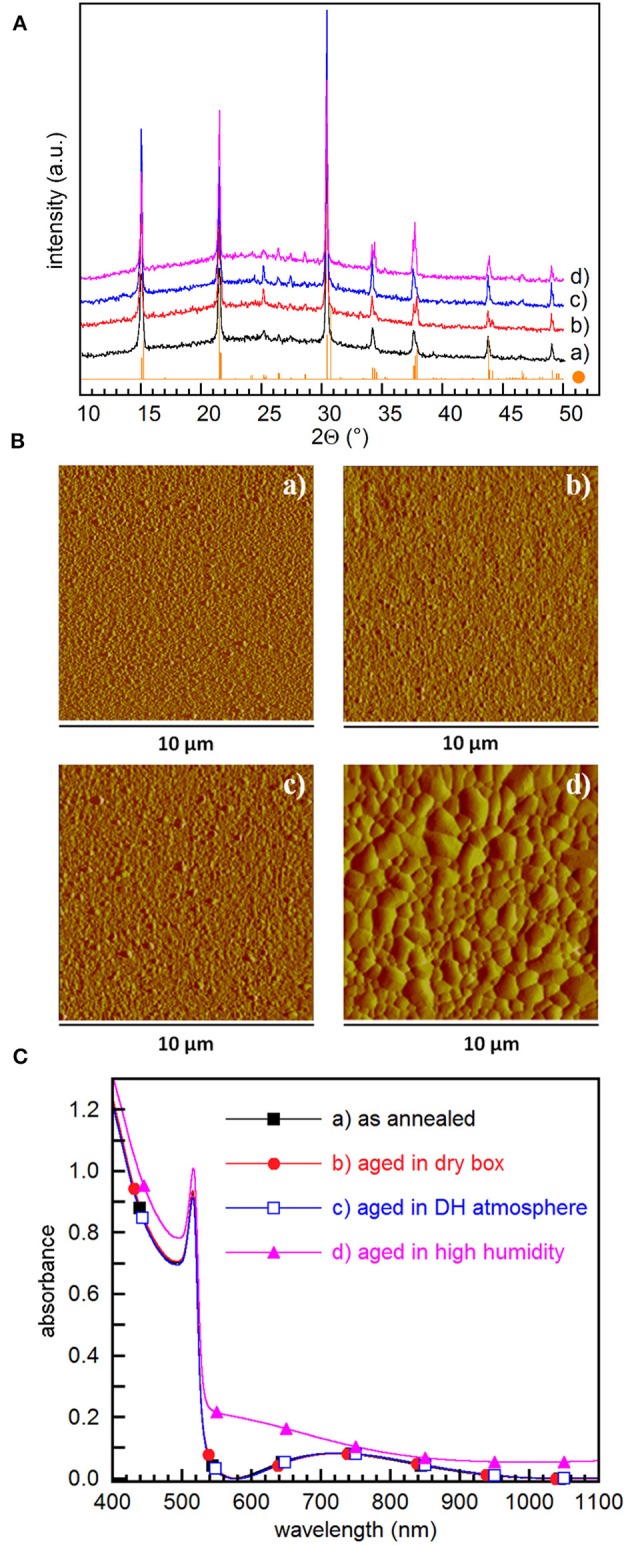
**(A)** XRD patterns, **(B)** AFM amplitude images, and **(C)** absorbance spectra of films converted into CsPbBr_3_ by 60 min annealing at 100°C: (a) soon after annealing, (b) after 50 days in dry box, (c) after 50 days in DH/atmosphere, and (d) after 5 days in 60%≤RH≤80% and 19 ≤ T ≤ 23°C. The CsPbBr_3_ reference XRD pattern (•) is also shown in **(A)** The corresponding AFM height images are reported in Figure S12.

We finally investigated the stability of prepared films when exposed to air, mainly to assess their suitability for the preparation of devices when carrying out the process into a glove box is not possible. To this aim, films converted into CsPbBr_3_ by overnight storage in DH atmosphere followed by annealing at 100°C for 60 min were aged for 50 days in either dry dark box or in DH atmosphere. XRD patterns show that aging does not make other phases appear since only CsPbBr_3_ signatures are detected, peak areas increasing on aging (Figure 5A) and being larger in films aged in DH atmosphere. AFM investigation (Figure 5B) points out that aging in dry air does not affect roughness that remains 7.63 nm, while aging in DH atmosphere results in an increased roughness of 16.7 nm.

Absorbance spectra confirm that films preserve their initial absorbance quality after 50 days aging (Figure 5C). Interestingly, even the exposure to humid air (60%≤RH≤80%) for 120 h does not make spurious phases appear (Figure 5A), although film surface becomes slightly hazy. Prolonging film exposure for 4 days at RH = 80% causes a minor XRD peak corresponding to the 022 reflection of CsPb_2_Br_5_ to appear at ~11.6°. This is in contrast with results reported for CsPbBr_3_ films obtained using a solution based two-step method (Di Girolamo et al., 2019), where exposure to RH = 80% for a few tens of minutes results in the degradation of CsPbBr_3_ by the formation of CsPb_2_Br_5_. We believe that the increased resilience of films flash-evaporated by SSTA comes from the improved film uniformity that minimizes the surface exposed to humidity, thus preventing CsPbBr_3_ from fast reacting with moisture. Absorbance spectra show that exposure to high humidity results in a reduction of the step height, with an increase of the background scattering likely due to the degradation of the surface (Figure 5C). Indeed, AFM measurements show that high humidity results in a dramatic enhancement of the grain size, with a surface roughness 10 times higher compared to the as converted films (Figure 5B). This is in fair agreement with investigations performed on methylammonium lead iodide-based films (You et al., 2014), where moisture induces grain boundary creep, which makes adjacent grains merge together. To the best of our knowledge, similar investigations are lacking for CsPbBr_3_ films, so that the mechanisms underlying film evolution due to moisture still need to be studied.

**Figure 6:**
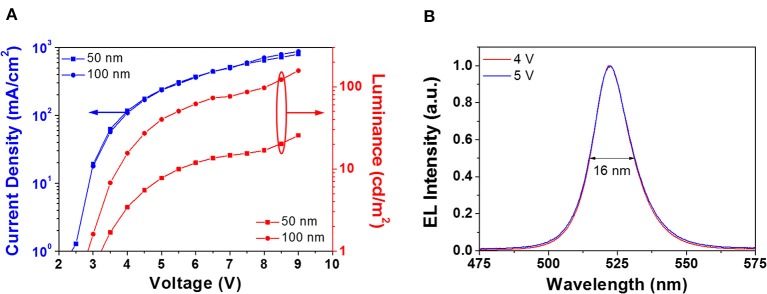
**(A)** Current densities (blue lines) and luminances (red lines) vs. voltage of 50 nm (squares) and 100 nm (dots) devices. **(B)** Electroluminescence spectra, peaked at 522 nm, measured at different applied voltages on 100 nm devices. The FWHM of the EL peak is also reported.

The above results show that smooth, compact, and pinhole-free CsPbBr_3_ films are obtained by either 24 h aging in DH atmosphere, or overnight storage in DH atmosphere followed by an annealing at 100°C for 60 min. With the aim of assessing the suitability of obtained CsPbBr_3_ films for planar devices, we prepared PeLEDs based on the latter conversion procedure because it yields the higher PL intensity (Figure 3). Two sets of PeLEDs were fabricated, having a perovskite-based active layer of either 50 nm (device A) or 100 nm (device B). In Figure 6A we plot the current density and luminance, as a function of the voltage, for both PeLEDs. The electrical behavior of these devices is quite similar, while a significant luminance increase can be observed for thicker CsPbBr_3_ films, since a maximum value of 25 and 150 cd/m^2^ was reached in device A and device B, respectively. It is worth noting that the turn-on voltage (@10 cd/m^2^) is about 3 V, a value very near to the thermodynamic limit given by the energy gap per unit charge, suggesting that the voltage drop due to interfaces does not have an important role in the light emission.

Interestingly, although the efficiency is rather low (Figure S13), Figure 6B shows that the normalized electroluminescence (EL) spectra of the devices correspond to the narrow EL emission of CsPbBr_3_, centered at 522 nm with a full width at half-maximum (FWHM) of 16 nm that equals the smallest value reported in the literature for green PeLEDs (Zhang F. et al., 2018). Besides, reported spectra obtained at different applied voltages exhibit no significant change, thus proving a high EL stability. It is worth noting that this is the first demonstration of halide perovskite films deposited by a single source vacuum technique exploited for PeLED fabrication. These results confirm that SSTA is potentially useful for the fabrication of planar hetero-structured devices.

## Conclusions

We investigated the preparation of films by single source thermal ablation of CsPbBr_3_ precursors, showing by XRD measurements that as-prepared films consist of a mixture of CsPbBr_3_, CsPb_2_Br_5_, and Cs_4_PbBr_6_ phases, due to vertical composition gradient following the partial decomposition of the precursor during evaporation. We showed that deposited films turn into pure CsPbBr_3_ phase in low humidity atmosphere, either spontaneously at room temperature or by mild thermal treatments. This conversion affects also film absorbance, whose spectra show an absorption onset at about 530 nm that is ascribed to CsPbBr_3_ and whose height increases with time until saturation is achieved. Morphology changes associated to the conversion into CsPbBr_3_ lead to polycrystalline films, whose features depend on the conversion procedure. Compact, smooth and pinhole-free films were achieved by aging in DH atmosphere at room temperature for 24 h or by annealing at 100°C after overnight aging in DH atmosphere. The suitability of obtained CsPbBr_3_ films for planar devices was assessed by fabricating PeLEDs with a very narrow green electroluminescence, thus confirming the potential of the obtained CsPbBr_3_ films. Reported results show how SSTA can be used profitably to achieve planar heterostructure devices based on all-inorganic halide perovskites.

## Data Availability Statement

The datasets generated for this study are available on request from the corresponding authors.

## Author Contributions

RM, MM, and AL designed the project. LN performed the AFM and TEM investigation. DC performed the SEM characterization. FMe performed the XRD characterization. PF contributed technically to the film deposition and XRD characterization. RM supervised the film deposition, contributed to the XRD characterization, performed the absorbance characterization, and wrote the first draft of the manuscript. AL performed the steady state and time resolved photoluminescence measurements. FMa and MM contributed in the fabrication and characterization of the electroluminescent devices. All authors contributed to the discussion and revision of the manuscript and approved the final version.

## Conflict of Interest

The authors declare that the research was conducted in the absence of any commercial or financial relationships that could be construed as a potential conflict of interest.
