# Supplementary material for: All-Inorganic CsPbBr3 Perovskite Films Prepared by Single Source Thermal Ablation
Source: Front Chem. 2020 Apr 21;8:313. doi: 10.3389/fchem.2020.00313 (PMC7186377; doi:10.3389/fchem.2020.00313)
Supplement: Supplementary file 1 [file Data_Sheet_1.PDF]

Supplementary Material

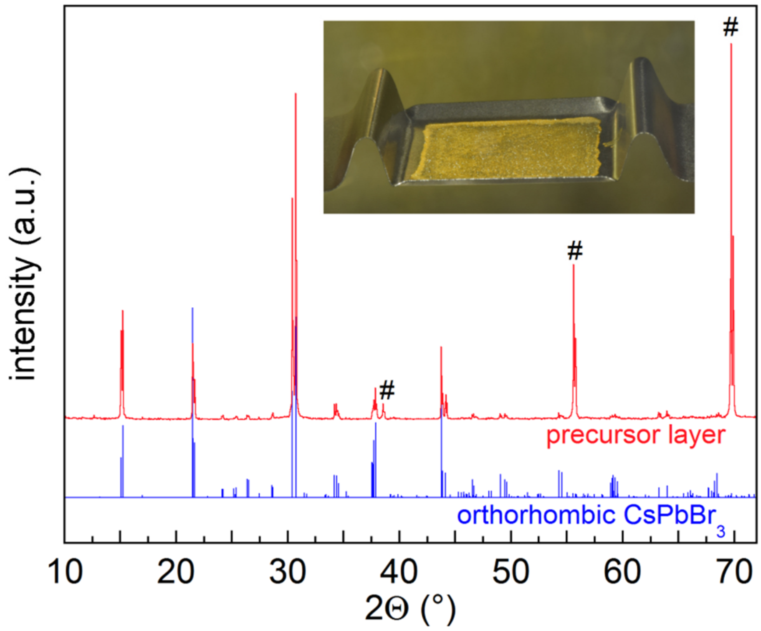

Figure S1 XRD pattern of the precursor layer deposited on a Ta foil (top pattern) compared to stick pattern of the standard powder diffraction pattern of bulk orthorhombic $CsPbBr_3$ (bottom pattern).(Rodová et al., 2003) Hashes indicate the Ta reflections. Inset shows the precursor in the Ta boat before evaporation.

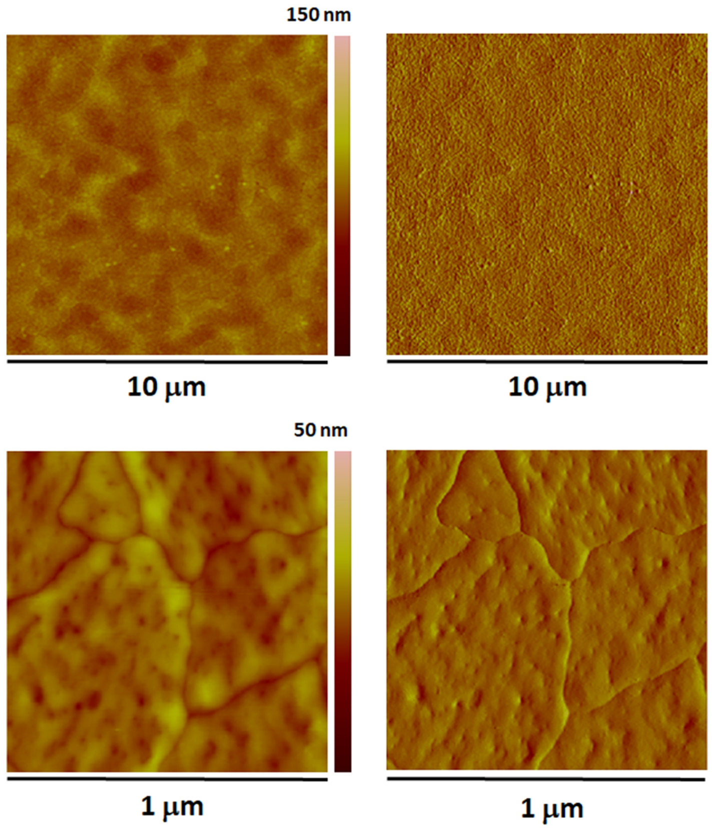

Figure S2. Typical AFM height (left) and amplitude (right) images taken on films soon after their extraction from the evaporation chamber.

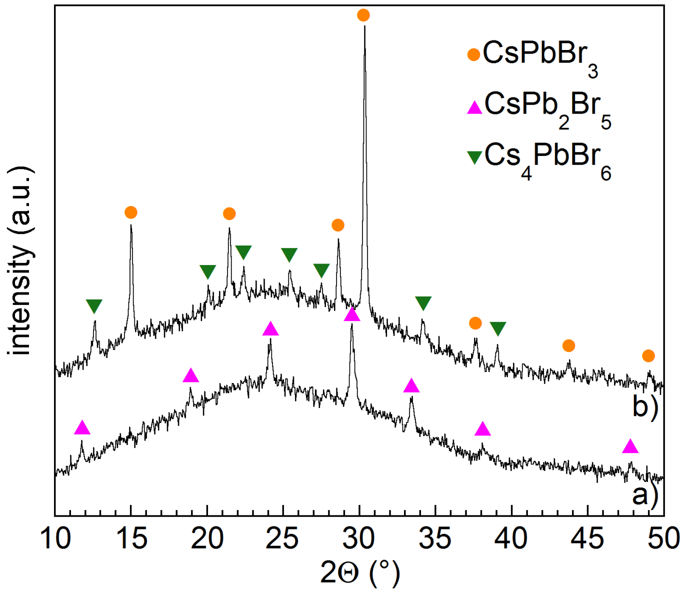

Figure S3 XRD patterns measured on films obtained by 150 W power after a) the initial evaporation phase (step 1) and b) the final evaporation phase (step 2). See Experimental section for details.

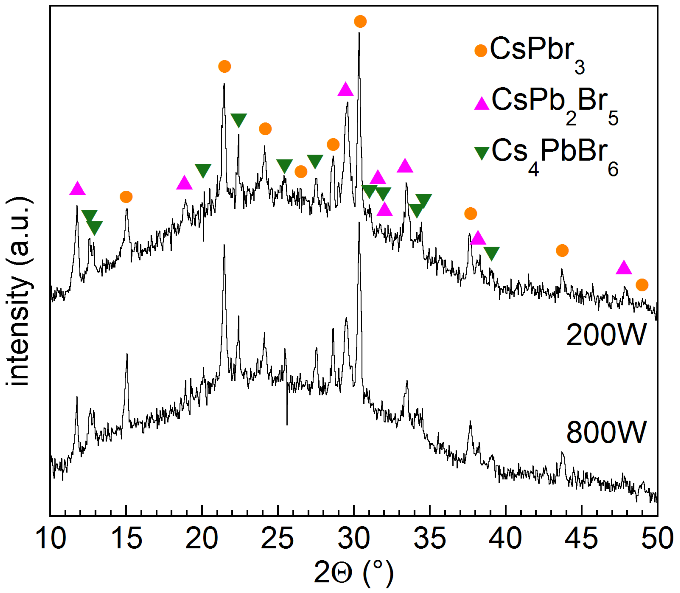

Figure S4 XRD patterns of films evaporated by using either 200 W or 800W power, as indicated.

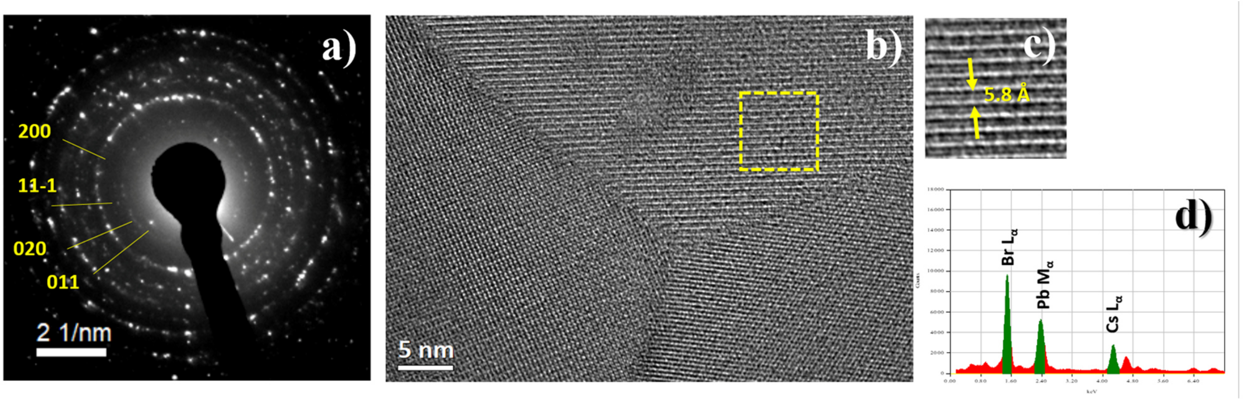

Figure S5 TEM analysis of a $CsPbBr_3$ films converted by 24h aging in DH atmosphere: a) SAED Pattern, b) HRTEM image of three adjacent grains, c) enlarged image of the square region marked in b), and d) EDXS spectrum.

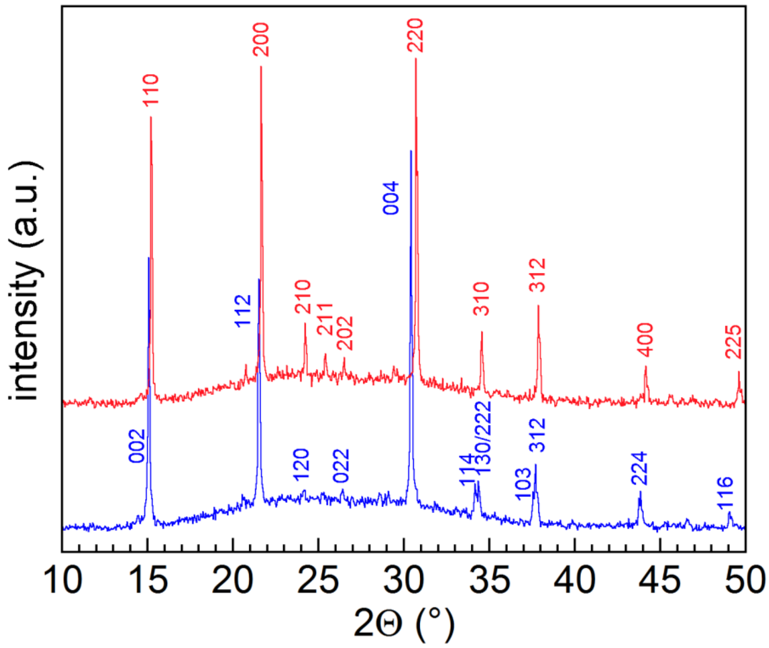

Figure S6 Typical XRD patterns of films converted by 24h aging in DH atmosphere (lower pattern) or by annealing at 200°C for 15 min (upper pattern).

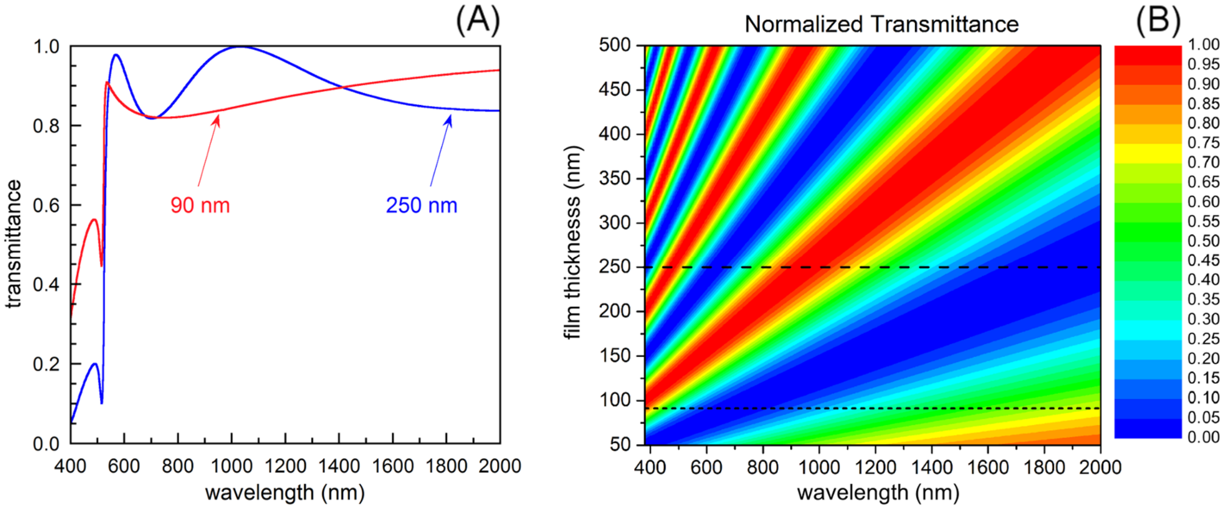

Figure S7 A) Measured transmittance curves for two different perovskite thicknesses, namely 90 and 250 nm. B) Color map of the simulated transmittance showing its normalized amplitude as a function of the radiation wavelength and the perovskite thickness.

Besides the transmittance drop at ~530 nm due to light absorption by $CsPbBr_3$, we found that the measured spectrum of the 90 nm sample exhibits only one minimum at ~750 nm, with a broad tail at higher wavelengths (Fig.S7A), while the 250 nm sample shows a narrow valley centered at ~700 nm

and wider one at ~2000 nm. This features were confirmed by exploiting an optical simulator to calculate the resonant wavelengths inside these samples (Fig.S7B), that we schematized as made of a glass substrate, a $CsPbBr_3$ film and air beyond it; the mean refractive index values used in the simulation were 1.5, 1.9 and 1.0, respectively. We considered a zero absorption condition and calculated the transmittance amplitude as a function of both the radiation wavelength and the perovskite thickness. The main features observed in the measured spectra in the 570-2000 nm wavelength range (Fig. S7B) were confirmed by simulated transmittance (Fig.S7B), the positions of the valleys in the calculated spectra differing slightly from those measured, probably due to i) the dispersion curve of the refractive index which was considered as constant in the calculation, and ii) the actual film thickness, as in the calculations we used the values measured at the edge of the sample by a stylus profilometer.
These results confirm that the measured transmittance trends are due to cavity effects.

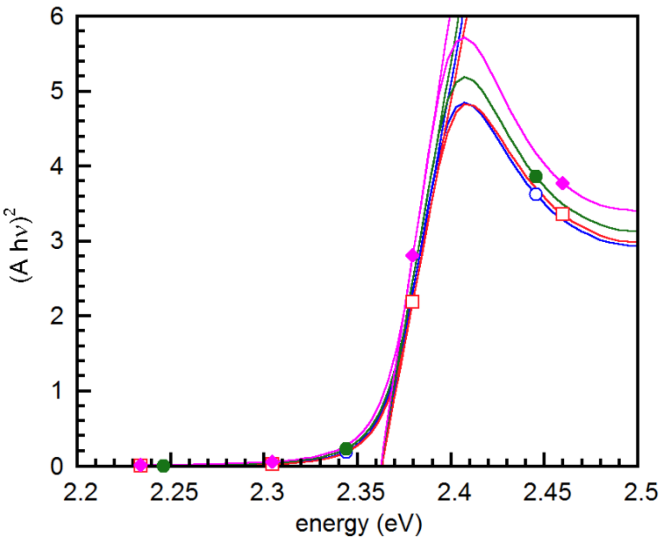

Figure S8 Tauc plot of films stored in DH atmosphere for 24h (○), stored overnight in air and then annealed at 100°C for 60 min (□), annealed at 150°C for 30 min (●), and annealed at 200°C for 60 min (◆).

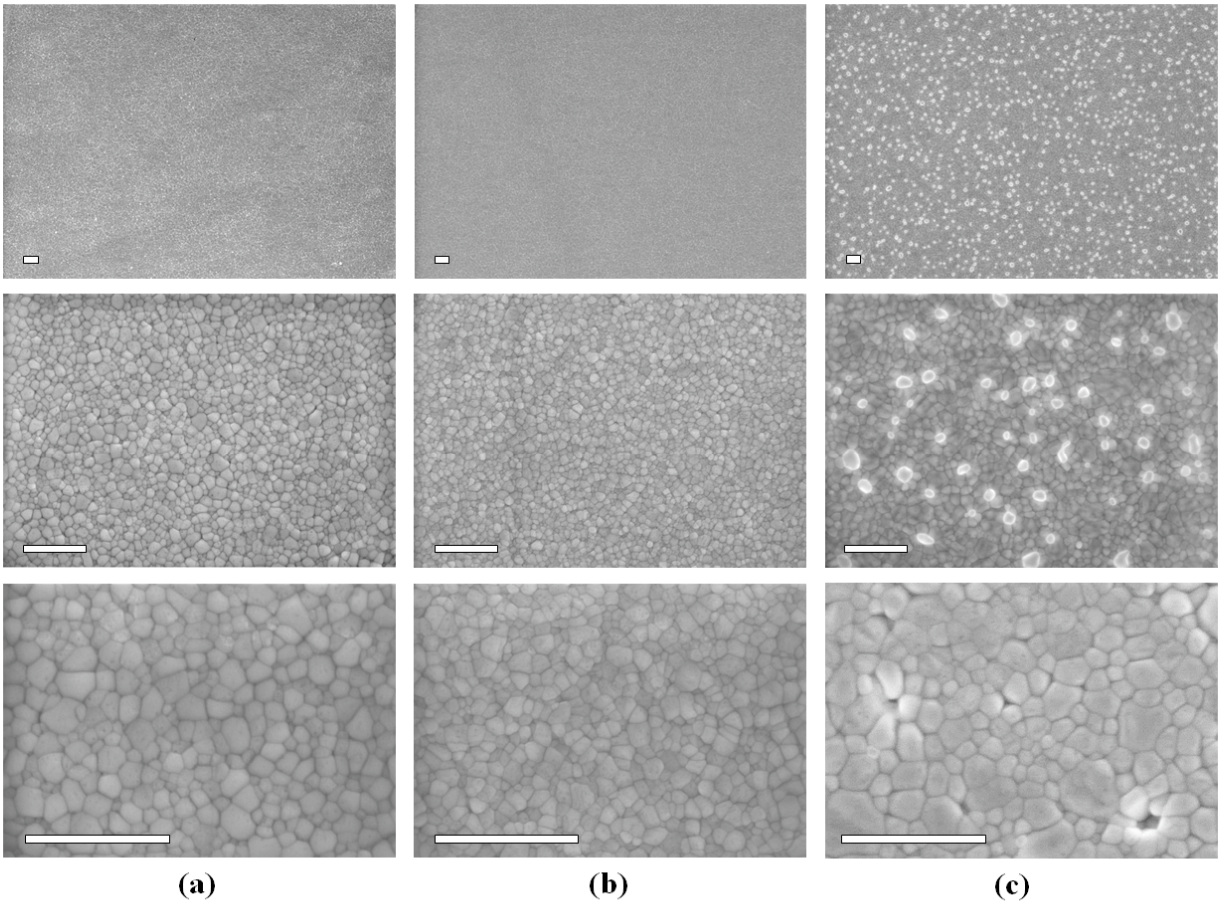

Figure S9 SEM images of films (a), aged in DH atmosphere for 24 h, (b) aged overnight in DH atmosphere and then annealed at 100°C for 60 min (c) annealed at 200°C for 60 min. Markers indicate 1 μm.

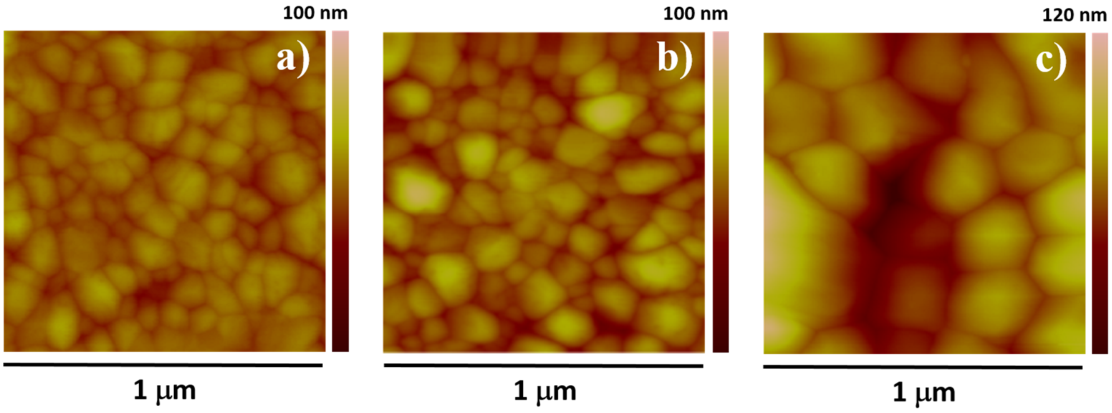

Figure S10 AFM height images of films a) aged in DH atmosphere for 24 h, b) annealed at 100 °C for 60 min after overnight storage in DH atmosphere, c) annealed at 200 °C for 60 min. The corresponding AFM amplitude images are reported in Fig. 4.

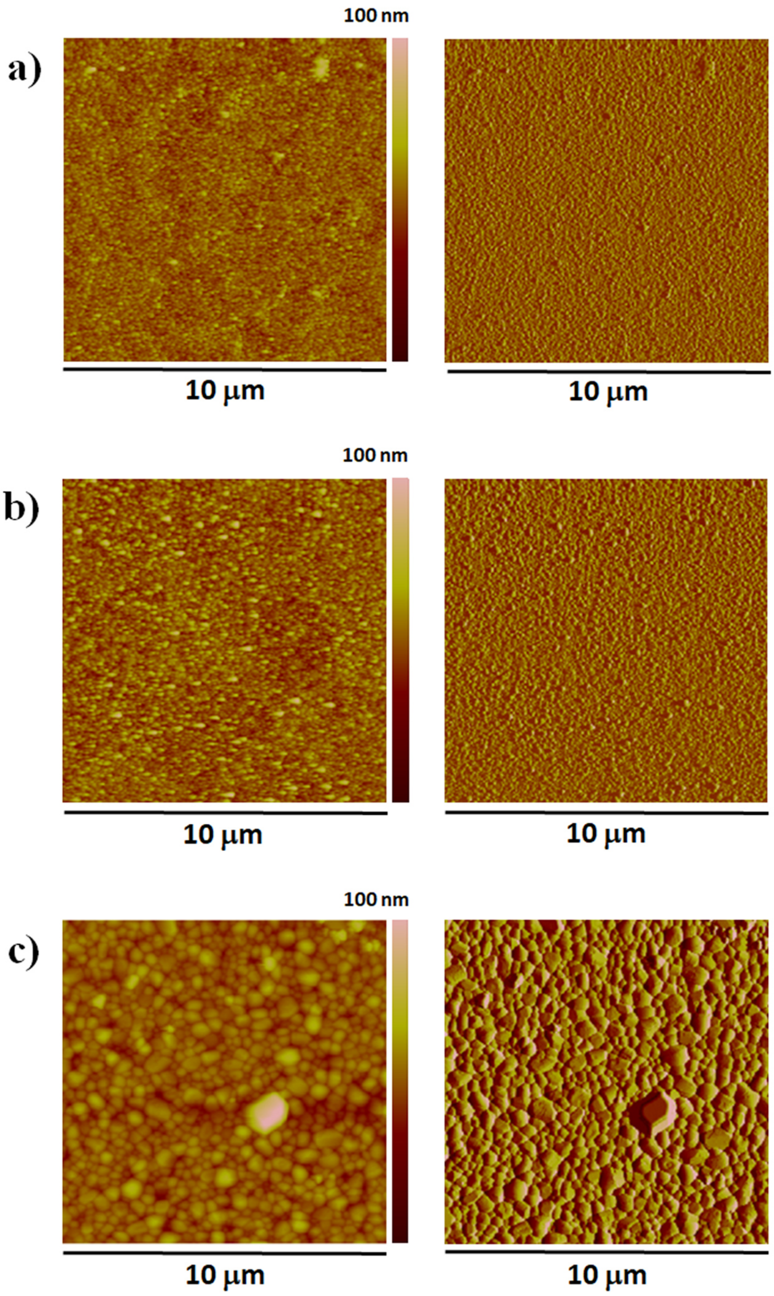

Figure S11 Low magnification AFM height (left) and amplitude (right) images of films a) aged in DH atmosphere for 24 h, b) annealed at 100°C for 60 min after overnight storage in DH atmosphere, c) annealed at 200°C for 60 min.

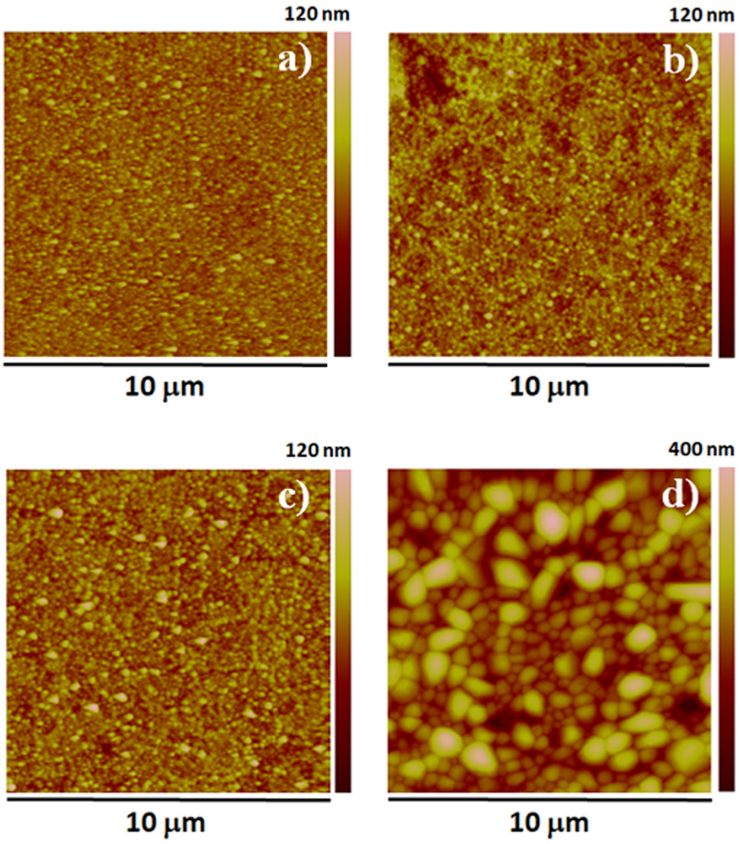

Figure S12 AFM height images of films converted into $CsPbBr_3$ by 60 min annealing at 100 °C: a) soon after annealing, b) after 50 days in dry box, c) after 50 days in DH\ atmosphere, and d) after 5 days in 60%≤RH≤ 80% and 19≤T≤ 23°C. Relevant amplitude images are shown in Fig. 5B.

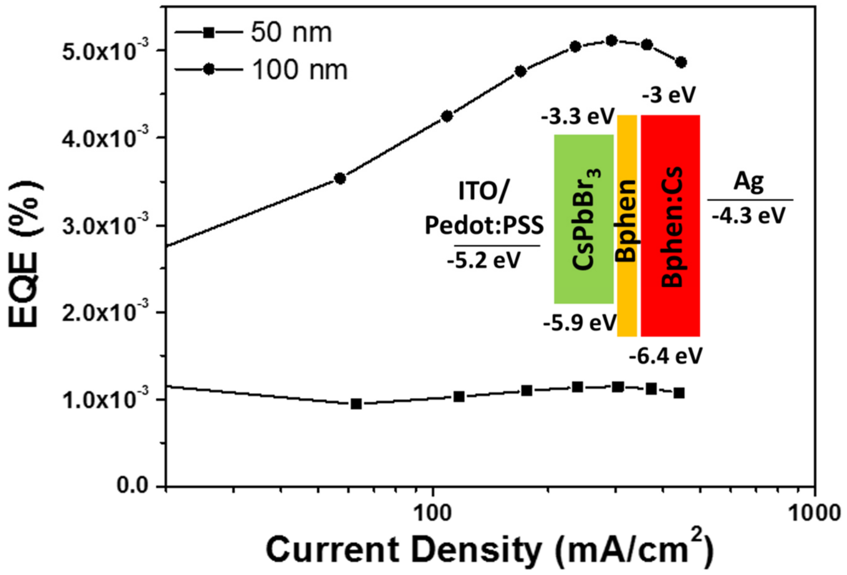

Figure S13 b) External quantum efficiency versus current density of 50 nm (squares) and 100 nm (dots) perovskite film devices. The inset shows the energy level diagram of the PeLED architecture.

The rather low EQE values (Fig.S13) are probably due to the process used for the conversion into $CsPbBr_3$, since for both 50 nm and 100 nm films we followed the procedure that gave the best results in 250 nm thick perovskite. Indeed, we observed that $CsPbBr_3$ conversion is faster for thinner films, so that the film evolution may depend on its thickness, which may affect properties such as non-radiative recombination through surface states, interfacial energetics and, to some extent, morphology. Then, the optimization of the device performance requires a detailed understanding of the thickness influence on film conversion, which is beyond the scopes of this work.

References

Rodová, M., Brožek, J., Knížek, K., and Nitsch, K. (2003). Phase Transitions in Ternary Caesium Lead Bromide. *J. Therm. Anal. Calorim.* 71, 667–673. doi:10.1023/A:1022836800820.
